# Modelling of transport processes: Theory and simulations

**DOI:** 10.1016/j.mex.2022.101966

**Published:** 2022-12-13

**Authors:** Ankita Gupta, Bipasha Pal, Akriti Jindal, Nikhil Bhatia, Arvind Kumar Gupta

**Affiliations:** a Department of Mathematics, Indian Institute of Technology, Ropar Rupnagar-140001, Punjab, India; b Department of Computer Science, Lakehead University, Thunder Bay, Ontario P7B 5E1, Canada

**Keywords:** Exclusion model, TASEP, Mean-field approach, Monte Carlo simulations, Mean-field approach

## Abstract

The transport processes, being a non-equilibrium system, have been a point of interest for physicists since many years revealing and explaining several unexpected effects. Such systems are often dealt with an archetypal model, known as totally asymmetric simple exclusion process, with two different types of boundary conditions: open and periodic. Moreover, these models are analyzed in two varieties of dynamics, random sequential and parallel updates, even at the micro level which play an important role in the global dynamics of the system. On contrary to the random sequential rule, the parallel updates introduce correlations in the system. Using theoretical and numerical methods in the framework based on mean-field approaches, the system properties are analyzed in both transient and steady state.

• Both the updating rules are realized using Monte Carlo simulations.
• In simplest form, mean-field approach ignores all the correlations and the results coincide with the random sequential update.
• Correlations are induced in the system due to parallel update, therefore, a cluster mean-field theory is also discussed to handle them.

## Graphical abstract

**Figure:**
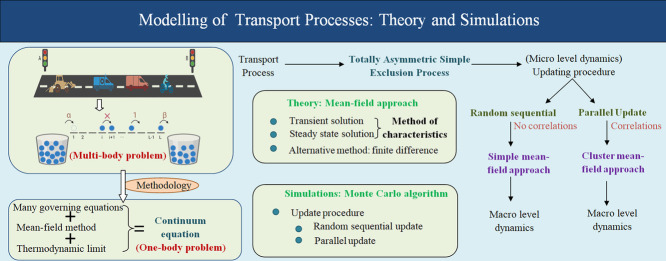

Specifications table

**Table:** 

Subject area:	Mathematical Modelling
More specific subject area:	*Transport process*
Method name:	Mean-field approach
Name and reference of original method:	*Phase diagram of one-dimensional driven lattice gases with open boundaries, Anatoly B Kolomeisky et al 1998 J. Phys. A: Math.Gen. 31 6911*
Resource availability:	*NA*

## Introduction

In the last few decades, the quest to comprehend the transport phenomena, both natural as well as man-made, has taken a considerable turn towards the study of statistical mechanics. It utilizes statistical and probabilistic methods, handles enormous populations, and makes use of approximations to understand the transport mechanisms that are frequently seen in many real-world processes [1]. These systems encompass a broad spectrum of topics, ranging from biological [2] to physical processes [3] and can be categorised into two groups: those that are far from equilibrium and those that are at or near equilibrium. Systems are said to be in equilibrium if they establish internal stability when isolated. It is said to be close to equilibrium if, after a little perturbation, it recovers to its equilibrium state. Equilibrium systems are only abstract idealizations since the equilibrium systems call for complete insulation to prevent interaction with the environment and the application of any net forces. Thanks to Gibbs and Boltzmann, these have been extensively researched and explored in the literature with a unified theory to understand such systems [4].

On the other hand, systems out of equilibrium have a state that is constantly changing throughout time as a consequence of external factors. In a long run, these systems may settle into a non-equilibrium steady state which, in contrast to the equilibrium counterpart, has a characteristic non-zero current. They frequently exhibit complicated behaviour as a result, and typically, no general conclusions can be drawn, such as those found in the principles of thermodynamics and statistical mechanics for systems in equilibrium. Therefore, numerous non-equilibrium systems that replicate real-world phenomena are studied using statistical mechanics, including movement of motor proteins on microtubules [5], [6], the behaviour of a colony of ants [7], [8], gel electrophoresis [9], [10], vehicular traffic [11], protein synthesis [12], [13], etc. These systems fall under a category termed driven-diffusive systems in which the particles can be either self-driven or field-driven [14], [15]. The self-driven systems have an internal driving force that is related to each particle and self-produced rather than being of external origin as in field-driven systems. The systems of our interest fall under the self-driven category, and the particles are a concept for many active systems, serving as a simplified as well as an abstract portrayal of the most significant dynamic activity of cells or entities [16].

A paradigmatic model that is frequently utilized for the class of self-driven diffusive systems is the totally asymmetric simple exclusion process (TASEP) [17], [18]. This model is described by a one-dimensional lattice having L sites and identical particles hopping unidirectionally (left to right) along the lattice. A maximum of one particle may occupy each site, or it may be empty. After each time step Δt, the particle positions are updated. Upon updating, a particle in site i hops to the right adjoining site i+1 with the unit rate provided it is empty; otherwise, it remains in site i. The lattice is facilitated with boundary conditions on its extreme ends which may either be open or periodic. The open boundaries are defined as follows. If the left boundary is vacant, a particle enters the lattice with the rate α. Furthermore, with rate β, a particle exits the lattice from the right boundary. These boundary conditions correspond to particle reservoirs at the left and right boundaries, which have a constant densities of α and 1−β, respectively [19]. On the other hand, periodic boundaries are characterized with the hopping of particles from right-most site to the left-most site only if the latter is vacant [20]. Since its inception, several modifications of TASEP have been studied, and by the means of these models and their generalized versions, it has been possible to comprehend various complex features such as delocalized and localized domain walls [21], [22], spontaneous symmetry breaking [23], phase separation [24], phase transitions [19], [25], [26], etc.

Several methodologies like Bethe ansatz [27], matrix ansatz [20], domain wall theory [21], [28], etc. have been developed over the years and the exact solutions to the TASEP model have been obtained. These exact approaches typically are difficult to generalize for incorporating more realistic dynamics corresponding to the system under consideration. However, approximate methods based on the mean-field approach [29], that may be used systematically to analyze many types of stochastic processes, can be utilized where exact solutions are not available. We mainly focus on the mean-field approach which relies upon ignoring few or all correlations in the system. This is due to the reasons that follow. Firstly, it significantly simplifies the analysis and still has been able to yield exact solutions to various models. Further, even if the simplistic model is modified to incorporate additional dynamics, the mean-field theory can be generalized to suit it with a relative ease.

To utilize the mean-field approach, the methodology involves approximating the governing equations and converting it to a partial differential equation. This reduces the many-body problem to a one-body problem, and thus locally on a macroscopic scale, the variables such as density, current, etc., can be defined. The disturbances in stochastic particle systems die out at large scales, and the system may be adequately characterised by deterministic evolution equations. This is achieved by examining the system in the thermodynamic limit [30], [31] which results in the partial differential equation that will be crucial to determine the macroscopic variables. Various approaches to obtain the solution of the system under consideration is to solve the partial differential equation using the numerical schemes, method of characteristics, etc. Alternatively, the partial differential equation can be studied in the steady state which gets rid of the time variable and reduces it to an ordinary differential equation that can be solved analytically to comprehend the non-equilibrium stationary properties.

Another important aspect of modelling the stochastic transport process is the updating procedure which must reflect the system properly. Many models above often assume a random-sequential update [32], [33], which means that the state of each particle is randomly updated after an exponentially distributed waiting time Δt. However, depending upon the system under consideration, it might not be feasible for various traffic systems, which need a parallel updating of vehicle positions [33], [34]. For instance, one might update the vehicle speeds simultaneously to reflect the driver’s reaction to a change in the traffic condition. The updating rule impacts the system on a microscopic level while the macroscopic dynamics remain unchanged which may introduce correlations in the system. These correlations, when neglected, may dilute the theoretical results. Thus, an appropriate mean-field technique must be used to capture the correlations in these scenarios.

The theoretical methods, in particular, the mean-field approaches, rely on approximations whose validity needs to be tested. One of the algorithms which efficiently captures the stochastic dynamics of the driven diffusive systems is the Monte Carlo simulation which is widely used in statistical mechanics [35]. In the upcoming sections, we also discuss this algorithm [36], for several update procedures in detail.

## Model description

The TASEP model consists of a one-dimensional lattice with L discrete sites labelled as i=1,2,⋯,L and identical particles. The sites i=2,3,⋯,L−1 are collectively called the bulk whereas the first and the last sites form the boundaries of the lattice. If the site i is occupied, and (i+1)th site is empty, then the particle moves to the latter with unit rate. Moreover, the particles obey the hardcore exclusion principle which ensures that a site is occupied by at most one particle. In addition to this, the lattice is also supplemented with certain boundary conditions which may strongly affect the stationary properties of the system. Based on the structure of the transport system, generally, two types of boundary conditions can be incorporated on TASEP, namely (i) open boundary conditions and (ii) periodic boundary conditions.

**Fig. 1:**
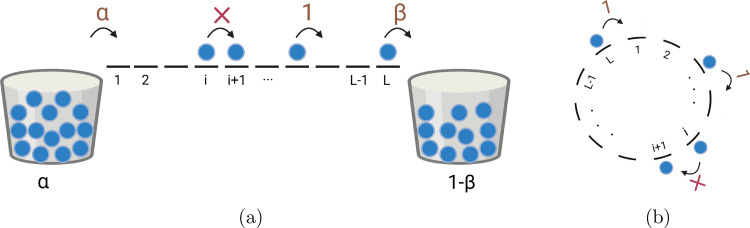
Schematic illustration of the TASEP system with different boundary conditions. (a) Open boundaries: particles can enter through the first site if empty with rate α, hop along the lattice to the neighbouring site from left to right with unit rate, and escape the lattice with rate β. (b) Periodic boundaries: particles from the last site enters the first site with a unit rate, provided the latter is empty [37].
• Periodic boundary conditions: A particle translocating on the lattice upon reaching the last site i=L hops to the first site i=1 with unit rate. This implies that i is substituted by i(modL) whenever i∈{1,2,⋯,L} and thus, the lattice takes the form of a ring. Since no particle can leave the lattice, the number of particles remains conserved in the system and serves as a parameter controlling the dynamics of the system. An illustration of this is shown in Fig. 1(b), where particles proceed in a clockwise direction.

• Open boundary conditions: In this scenario, the particles can enter the lattice from the left boundary with the rate α provided the first site is empty, and leave the lattice from the right boundary with rate β. Such conditions imply that the number of particles on the lattice is not conserved and the properties of the system are controlled by the boundary dynamics. To realise these boundary conditions, the left and the right boundaries of the lattice are connected to reservoirs having constant densities, α and 1−β, respectively, as depicted in Fig. 1(a).

**Fig. 2:**
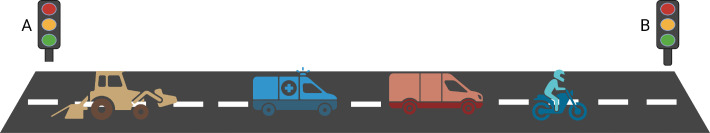
Schematic diagram for a segment of road with two traffic lights A and B where vehicular traffic is regulated through these lights, demonstrate a TASEP model with open boundary conditions [37].

Being a paradigm in the elementary stochastic models, it is found that TASEP is competent enough to mimic several physical and biological transport phenomena. For instance, the vehicular movement in a part of a one-way road having two traffic lights, A and B, situated at its ends can be modelled using the TASEP with open boundaries [9], [20], [21], [25]. A cartoon figure of this process is provided in Fig. 2. The segment of the road and the vehicles can be represented as a finite discrete lattice and the particles, respectively, and move forward while obeying some preassigned rules. Traffic lights A and B control the entry and exit of vehicles on the road which correspond to the open boundary conditions. Physically, it is clear that no two vehicles can occupy the same position on the road which justifies the hardcore exclusion principle of TASEP. Also, the movement of motor proteins along the microtubules in a eukaryotic cell is an example of biological transport which can be likewise emulated by TASEP with open boundaries [16]. Such models acquire many interesting non-equilibrium characteristics and effectively explain the complex dynamics of the transport phenomena [2]. TASEP can act as a minimal model for any stochastic process where the transport is governed by a certain set of rules and falls under non-equilibrium systems.

In the next section, we discuss several methods to update the lattice sites in detail and address their impact on the stationary properties of the system.

## Updating rules

The updating procedure serves as an essential aspect of mimicking transport phenomena and must properly demonstrate the dynamics of the system under consideration. These rules crucially affect the system on a macroscopic scale whereas the microscopic kinetics are unaltered. Broadly, there exist four main classes of updating rules:

• Random sequential update
• Sublattice parallel update
• Ordered sequential update
• Parallel update

A detailed description of all these updating rules can be found in the reference [33]. Many models involving cellular transport frequently consider the random sequential update procedure, whereas the vehicular traffic models may require the parallel updating rule. Owing to the aforementioned reasons, in this article we focus only on random sequential and parallel update rules, and these are described in detail as follows:

1. Random sequential update rule: It is a continuous time updating rule where in each time step, at first a site i is chosen randomly and, then gets updated according to its allocated dynamics as shown in Fig. 3. More specifically, if site i ∈{1,2,⋯,L−1} is occupied, the particle hops forward with a unit rate to the site (i+1) provided the target site is vacant. For the open boundaries, if the first site is selected and is empty, then a particle gets injected with a rate α whereas if the last site is chosen and is occupied, then the particle leaves the lattice with a rate β. In the case of periodic boundaries, if site L is chosen and found to be occupied, then the particle moves to the first site in case the latter is empty. Apart from the above-mentioned updating conditions, no change occurs in the position of the particles. Since this update rule contains high randomness, it is suited to mimic the intracellular transport where the forward movement of motor proteins depends on the hydrolysis of ATP. This process itself is stochastic and may not take place simultaneously for all motor proteins.

**Fig. 3:**
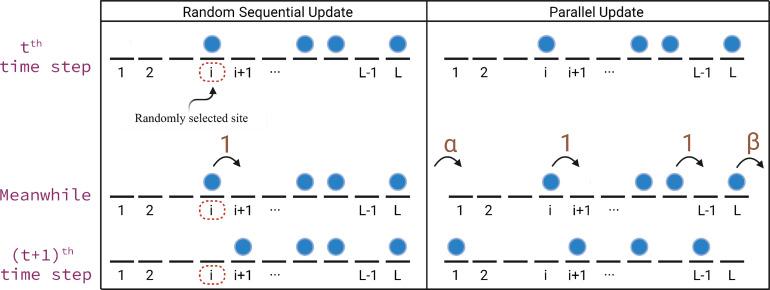
The diagram depicts the transition from the tth time step to the (t+1)th time step for the TASEP supplemented with open boundaries for different update procedures. (a) Random sequential update: Here a site i is randomly selected and updated at the (t+1)th time step according to the possible dynamics that can occur at this site. (b) Parallel update: All sites are updated simultaneously based on the dynamics possible at the corresponding site [37].
2. Parallel update rule: This is a discrete-time updating rule in which, at each time step, all the sites are updated simultaneously. For open boundaries, injection at site 1 with a rate α, removal from the site L with a rate β, and hopping of particles in the lattice with a rate 1 are implemented in each time step. The same is depicted in Fig. 3. Analogous updates are performed in case of periodic boundaries. Undoubtedly, these updating rules model the behavior of traffic flow and pedestrian flow, where all vehicles or pedestrians move synchronously in one time step.

## Mathematical treatment: Master equations

Let the state of each site for the discrete lattice be denoted by a particle occupation number $\tau_{i}$ where each $\tau_{i}$ is a binary variable defined as

$$\begin{array}{cc} \tau_{i} & =\left\{ \begin{array}{cc} 1; & \;\text{if}\;\text{site}\;i\;\text{is}\;\text{occupied}\;\text{by}\;\text{a}\;\text{particle}\;\\ 0; & \;\text{if}\;\text{site}\;i\;\text{is}\;\text{empty.}\; \end{array}\right. \end{array}$$

The configuration of the system at any time t is denoted by $\tau \left(t\right)=(\tau_{1}\left(t\right),\tau_{2}\left(t\right),\ldots ,$ $\tau_{L}\left(t\right))$. The time evolution of the probability P(τ,t) to find the system in configuration $\left(\tau_{1},\tau_{2},\ldots ,\tau_{L}\right)$ is determined by the master equation as given below:

(1) $$\frac{\partial P(\tau ,t)}{\partial t}=\underset{Gain}{\underset{︸}{\sum_{\tau^{\prime }\neq \tau }\omega \left(\tau^{\prime }\to \tau \right)P\left(\tau^{\prime },t\right)}}-\underset{Loss}{\underset{︸}{\sum_{\tau^{\prime }\neq \tau }\omega \left(\tau \to \tau^{\prime }\right)P\left(\tau ,t\right)}},$$

where $\omega \left(\tau^{\prime }\to \tau \right)$ and $P\left(\tau^{\prime },t\right)$, respectively represents the transition rates from state $\tau^{\prime }$ to τ and the probability for the system to be in configuration $\tau^{\prime }$. The first term on the right-hand side of Eq. (1) denotes the gain term by possible transitioning of the configurations from $\tau^{\prime }$ to τ, whereas the second term deals with the probability of departing from configuration τ.

Let $\tau \left(0\right)=\left(\tau_{1}\left(0\right),\tau_{2}\left(0\right),\ldots \ldots ,\tau_{L}\left(0\right)\right)$ be any initial configuration of the system, then the time evolution of $\langle \tau_{i}\left(t\right)\rangle \forall i$ for open boundary conditions can be computed from the following master equations:

(2) $$\frac{d\langle \tau_{1}\rangle }{dt}=J_{enter}-J_{1,2},$$

(3) $$\frac{d\langle \tau_{i}\rangle }{\mathrm{dt}}=J_{i-1,i}-J_{i,i+1},\;1<i<L,$$

(4) $$\frac{d\langle \tau_{L}\rangle }{dt}=J_{L-1,L}-J_{exit},$$

where 〈⋯〉 denotes the statistical average. Here, $J_{i,i+1}$ describes the flux or current which is the average number of particles passing from site i to i+1 per unit time and is given by

(5) $$J_{i,i+1}=\langle \tau_{i}\left(1-\tau_{i+1}\right)\rangle .$$

The term $J_{enter}$ ($J_{exit}$) represents the current entering (exiting) from the first (last) site and is expressed as

(6) $$J_{enter}=\alpha \langle 1-\tau_{1}\rangle ,\;J_{exit}=\beta \langle \tau_{L}\rangle .$$

Eq. (2) describes the change in density at the first site and is given by the difference in the average flow of particles to site 1 from the left reservoir with rate α (gain term) and out from site 1 to site 2 with the rate 1 (loss term). Similarly, right-hand sides of Eqs. (3) and (4) represent the difference of gain and loss terms for evolution in $\langle \tau_{i}\rangle$. For periodic boundary conditions, the master equation given by Eq. (3) is modified such that for i=1, $\tau_{i-1}=\tau_{L}$ and when i=L, $\tau_{i+1}$ is replaced with $\tau_{1}$.

We have now developed a frame for comprehending the dynamic properties of the system imposed with different boundary conditions and the updating rules described above. In the subsequent sections, we analyzed the system using a suitable theoretical approach and focus on how these updating rules affect the stationary state properties of the system with open and periodic boundary conditions.

## Analysis of TASEP with open boundary conditions

To scrutinize the system, we need to solve the Eqs. (2)–(4) that involves two-point correlators. Our motive is to obtain the explicit values for $\langle \tau_{i}\rangle \;\forall i$ which is intractable in the present form. Therefore, we utilize an approximation, known as mean-field theory (MFT), to break it into smaller correlators [29].

### Mean-field theory

This approximation method ignores all kinds of correlations between any two sites i.e. presence of a particle at one site doesn’t affect the presence of a particle at another site. The method being the simplest form of mean-field approximation is referred to as naive mean-field approximation. Thus the statistical average of the two sites is equal to the product of their individual averaged effect i.e.

(7) $$\langle \tau_{i}\tau_{j}\rangle =\langle \tau_{i}\rangle \langle \tau_{j}\rangle .$$

Utilising mean-field framework, Eqs. (2)–(4) can be written as

(8) $$\frac{d\rho_{1}}{dt}=\alpha \left(1-\rho_{1}\right)-\rho_{1}\left(1-\rho_{2}\right),$$

(9) $$\frac{d\rho_{i}}{dt}=\rho_{i-1}\left(1-\rho_{i}\right)-\rho_{i}\left(1-\rho_{i+1}\right),$$

(10) $$\frac{d\rho_{L}}{dt}=\rho_{L-1}\left(1-\rho_{L}\right)-\beta \rho_{L},$$

where $\langle \tau_{i}\rangle \equiv \rho_{i}$ denotes the average particle density at each site. The corresponding solution of the above equations can be obtained by coarse-graining the discrete lattice with lattice constant ϵ=1/L to a continuum limit and rescaling the time as t=t/L. Applying Taylor’s series expansion for $\rho_{i\pm 1}$ and retaining the terms up to second-order reduces Eq. (9) to

(11) $$\frac{\partial \rho }{\partial t}=\frac{\partial }{\partial x}\left(\frac{\epsilon }{2}\frac{\partial \rho }{\partial x}-\rho \left(1-\rho \right)\right),$$

where x=i/L,0<x≤1 denote the rescaled position variable and ρ describes the average particle density. The boundary conditions will be later dealt with accordingly, as and when required in the subsequent sections. Note that the subscript i was dropped because the lattice is free from inhomogeneity of any kind. Now, in the thermodynamic limit, L→∞ (or ϵ→0), Eq. (11) can be rewritten as

(12) $$\frac{\partial \rho }{\partial t}+\frac{\partial J(\rho )}{\partial x}=0,$$

where

(13) J(ρ)=ρ(1−ρ),

expresses the current-density relationship. We first analyze the above partial differential equation (Eq. (12)) for transient solutions and then proceed further to examine the stationary properties of the system.

### Transient solution

We commence by investigating the shock wave and rarefaction wave that might be produced by the initial value problem

(14) $$\begin{array}{cc} & \frac{\partial \rho }{\partial t}+\frac{\partial J(\rho )}{\partial x}=0,\\ & \rho (x,0)=g(x), \end{array}$$

where the initial density step g(x) is described as

(15) $$g\left(x\right)=\left\{ \begin{array}{cc} \rho^{-}; & x<0\\ \rho^{+}; & x>0 \end{array}\right.$$

with $\rho^{-}=\alpha$ and $\rho^{+}=1-\beta$. The choice of such $\rho^{-}$ and $\rho^{+}$ will be discussed in the next subsection. The initial value problem defined by Eqs. (14) and (15) is known as Riemann’s problem where the initial data consists of a single discontinuity. While trying to solve Eq. (14) by the method of characteristics, we arrive at the following characteristic equations:

(16) $$\frac{dt}{ds}=1,$$

(17) $$\frac{dx}{ds}=1-2\rho ,$$

(18) $$\frac{d\rho }{ds}=0.$$

The corresponding initial condition can be stated as

(19) t(ζ,0)=0,

(20) x(ζ,0)=ζ,

(21) ρ(ζ,0)=g(ζ).

It is straightforward to obtain the solution to the characteristics equations as

(22) t=s,

(23) ρ=g(ζ),

(24) x=(1−2g(ζ))s+ζ.

Clearly, an implicit solution to the initial value problem Eq. (14) is given by

(25) ρ(x,t)=g(x−(1−2ρ)t).

Since $\frac{d\rho }{ds}=0$, it asserts that ρ is constant along the projected characteristics curve provided by Eq. (24).

When ζ<0, the initial condition given by Eq. (15) implicates $g\left(\zeta \right)=\rho^{-}$. This further yields $x=(1-2\rho^{-})t+\zeta$ and ρ takes the value $\rho^{-}$ which remains constant along these curves. Similarly, for ζ>0, the projected characteristics are given by $x=(1-2\rho^{+})t+\zeta$ throughout which ρ is equal to $\rho^{+}$.

The behaviour of the system depends predominantly on the values of $\rho^{-}$ and $\rho^{+}$, therefore, we categorize our further study according to the sign of $\rho^{-}-\rho^{+}$ i.e., α−(1−β).

**Case**(i)α−(1−β)<0: Let us inspect the characteristics curves given by

(26) $$x=\left\{ \begin{array}{cc} (1-2\alpha )t+\zeta ; & \zeta <0\\ (1-2(1-\beta ))t+\zeta ; & \zeta >0. \end{array}\right.$$

Note that the slope of the characteristics curve for ζ<0 is greater than the slope of the curve when ζ>0, i.e.,

(27) 1−2α>1−2(1−β).

**Fig. 4:**
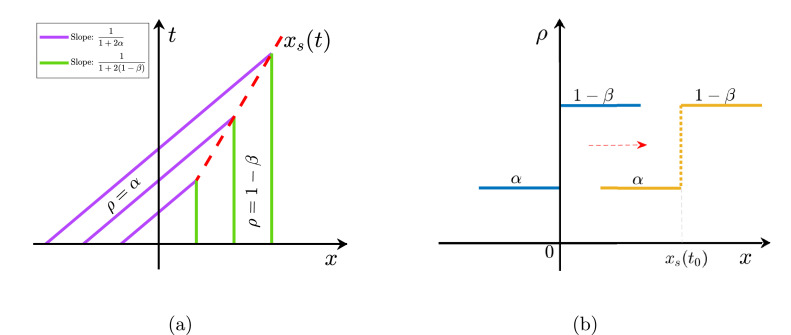
(a) Intersecting characteristic curves for the case when α<1−β implies the presence of a shock wave. The solid green and purple lines represent the characteristics curves x=(1−2α)+ζ and x=(1−2(1−β))+ζ, respectively. The dotted red line corresponds to the points of intersection of the two curves. (b) Time evolution in the particle density from Riemann condition to a shock wave at time $t_{0}>0$. The point $x_{s}\left(t_{0}\right)$ gives the position of the shock.

The two characteristics curves when plotted in t−x plane intersect each other, as seen in Fig. 4(a), and consequently, no classical solution to the problem defined by Eq. (14) can be achieved. Instead, a weak solution may be obtained which can be piece-wise differentiable function and satisfies the Rankine-Hugoniot jump condition. We are interested in calculating a curve $x=x_{s}\left(t\right)$ such that ρ takes the value α to the left of the curve and 1−β to the right of the curve. Precisely, this implies that the solution is classical on both sides of the discontinuity located at the abscissa $x_{s}\left(t\right)$. This solution is known as the shock wave and the speed of the shock s must satisfy the Rankine-Hugoniot jump condition

(28) $$s=\frac{J\left(\rho^{+}\right)-J\left(\rho^{-}\right)}{\rho^{+}-\rho^{-}}.$$

Utilizing the expression for current, we obtain the speed of the shock as

(29) $$s=1-\rho^{+}-\rho^{-}=\beta -\alpha .$$

Thus, the initial density step merely translates with time as depicted in Fig. 4(b) and the solution reads

(30) $$\rho \left(x,t\right)=\left\{ \begin{array}{cc} \alpha ; & x<x_{s}\left(t\right)\\ 1-\beta ; & x>x_{s}\left(t\right) \end{array}\right.$$

where $x_{s}\left(t\right)=st$ is the desired curve.

**Fig. 5:**
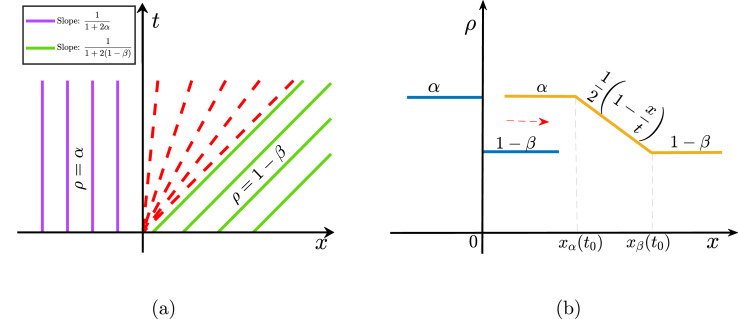
(a) Characteristic curves for a rarefaction wave for α>1−β. Solid green and purple lines correspond to curves along which ρ takes the value α and 1−β, respectively. Dotted red lines represent the region of the rarefaction wave. (b) Time evolution in the particle density from Riemann condition to a rarefaction wave solution.

**Case**(ii)α−(1−β)>0: In contrast to the previous situation, the characteristics curve does not show any crossing. In fact, we have a region where there is not enough information to obtain a classical solution (see Fig. 5(a)). Here, the solution is classical on both sides of rarefaction located between the curves x=(1−2α)t and x=(1−2(1−β))t along which ρ=α and ρ=1−β are constant solutions, respectively. For such a rarefaction wave, the solution is defined as follows:

(31) $$\rho \left(x,t\right)=\left\{ \begin{array}{cc} \rho^{-}; & x<J^{\prime }\left(\rho^{-}\right)t\\ f(x/t); & J^{\prime }\left(\rho^{-}\right)t<x<J^{\prime }\left(\rho^{+}\right)t\\ \rho^{-}; & J^{\prime }\left(\rho^{+}\right)t<x \end{array}\right.$$

where $J^{\prime }=\frac{dJ}{d\rho }$ and $f\equiv {\left(J^{\prime }\right)}^{-1}$. Using the expression for J from Eq. (13), the solution for the rarefaction part is obtained as

(32) $$f\left(z\right)=\frac{1}{2}\left(1-z\right).$$

Finally, the initial density step relaxes into a rarefaction wave as presented in Fig. 5(b) and the solution reads

(33) $$\rho \left(x,t\right)=\left\{ \begin{array}{cc} \alpha ; & x\leq x_{\alpha }\\ \frac{1}{2}\left(1-\frac{x}{t}\right); & x_{\alpha }\leq x\leq x_{\beta }\\ 1-\beta ; & x_{\beta }\leq x \end{array}\right.$$

where $x_{\alpha }=\left(1-2\alpha \right)t$ and $x_{\beta }=(1-2\left(1-\beta \right))t$.

### Steady state properties

So far, we have discussed the transient solutions that describe the time-dependent evolution of the system taken into account. In many situations, such as vehicular flow and movement of motor proteins, the steady state properties are crucial in understanding the complex dynamics of the system. However, after a long time, these systems can achieve a stationary state that is reflected by non changing properties with respect to time. One of the key features of such a non-equilibrium system is the presence of a non-zero particle current. Now, we perform a comprehensive study to examine the stationary properties, in particular, the particle density at each site.

To investigate the behaviour of the system at steady state, we study Eqs. (30) and (33) as t→∞. For further analysis, we consider α,β∈[0,1] and thoroughly discuss the asymptotic nature of the system for a large time. Since in the TASEP model, we are restricted to the domain x∈[0,1] due to the rescaling of the lattice in subsection 5.1. Therefore, we shift our point of discontinuity in the initial conditions from x=0 to x=0.5 and the shock or rarefaction solutions can be converted accordingly.

**Fig. 6:**
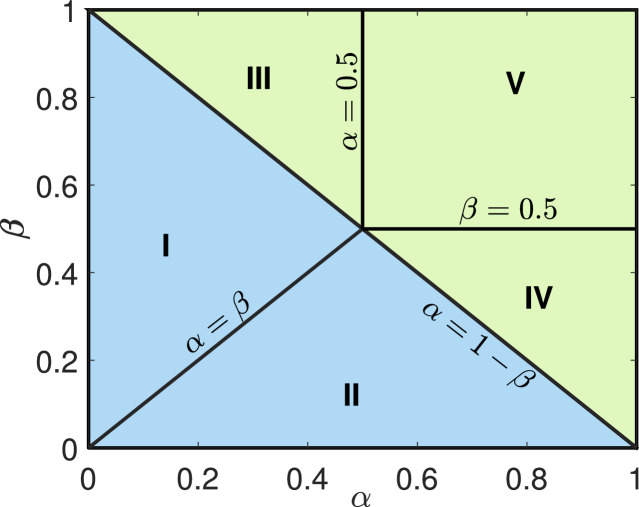
Phase diagram for the system with open boundary conditions is presented in α−β plane. The blue-colored area (region I and II) corresponds to the portion where the particle density is a shock wave solution whereas the green area (region III, IV, and V) represents the region where rarefaction wave solution sustains.
• α>1−β: The solution in this scenario is of rarefaction type which we have explained in subsection 5.2. For β>1/2 and α<1/2 (region III in Fig. 6, we have 1−2α>0 and 1−2(1−β)>0 implying that the rarefaction waves are driven out through the right boundary, eventually leading to the constant solution α as t→∞. Similarly, for β<1/2 and α>1/2 (labelled as region IV in Fig. 6, both 1−2α and 1−2(1−β) are negative quantities and the steady state particle density is thus given by 1−β.
  Finally, the unexplored region is α>1/2 and β>1/2 that is described by region V in Fig. 6. Here, the points $x_{\alpha }+0.5$ and $x_{\beta }+0.5$ translocate towards left and right with evolution in time, leading to the expansion of rarefaction waves, and the density is completely defined by
  (34) $$\rho \left(x,t\right)=\frac{1}{2}(1-\frac{x}{t}),$$
  which in the limiting case of t→∞ reduces to the constant density solution 1/2.

• α<1−β: In this region, the solution corresponds to a shock wave as discussed in subsection 5.2. Here, the speed of the shock is given by β−α which changes its sign about α=β. For β>α (denoted by region I in Fig. 6), the shock travels towards the right due to the positive speed where it eventually dissipates and leads to the stationary particle density given by α. With similar reasoning, for β<α represented by region II in Fig. 6, the shock speed is negative and as t→∞, the density solely depends upon the right boundary condition which is equal to 1−β.

To summarize, three different stationary solutions to the particle densities have been obtained in the α−β parameter space. These densities are given by α, 1−β, and 1/2 which are designated as low density (LD), high density (HD), and maximal current (MC) phases, respectively. Now, we discuss the properties of each phase in detail.

#### Low density phase

The bulk density in this phase is entirely described by the solution ρ=α. Due to the properties being controlled by the entry rate, this phase is also known as the entrance-dominated phase. It can be readily concluded that the existence condition of this phase is given by

(35) α<min{β,1/2}.

Moreover, the above equation implies that the density in this phase remains less than 1/2, hence it is designated as low density phase. Another inference that can be drawn from Eq. (35) is that the entrance rate α always remains less than the exit rate β, therefore it is more likely that the particles are exiting the lattice faster than their entry.

#### High density phase

In this phase, the bulk density is expressed as ρ=1−β, and is regulated by the exit rates, this phase is referred to as the exit-dominated phase. The existential criteria can be easily derived and is given as

(36) β<min{α,1/2}.

The conditions β<α imply that the particles will enter faster as compared to leaving. Therefore, the presence of a huge number of particles on the lattice leads to a density greater than 1/2, and is identified as high density phase

#### Maximal current phase

For this situation, the density profile becomes independent of the entry-exit rates and always takes the value 1/2. The existence of such a phase requires

(37) min{α,β}>1/2.

The current attains its maximum value which is given by 1/4, and hence, this phase is termed maximal current. This corresponds to the extreme point of J and coincides with the solution of $\frac{dJ}{d\rho }=0$. Moreover, α and β both are greater than 1/2, therefore, particles enter and leave the lattice quickly.

**Fig. 7:**
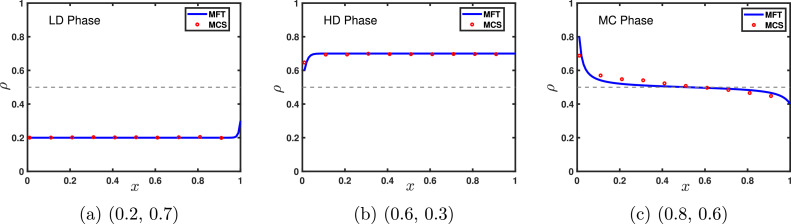
Typical density profiles for the system with open boundaries and random sequential update rule of a (a) LD, (b) HD, (c) MC phase for (α,β) provided in sub captions. In all figures, solid lines are the results obtained using mean-field approximation and symbols denote Monte Carlo simulations for L=100. The gray dotted line is drawn at the value 1/2 to visualize the particle density compared to the density in the MC phase.

**Fig. 8:**
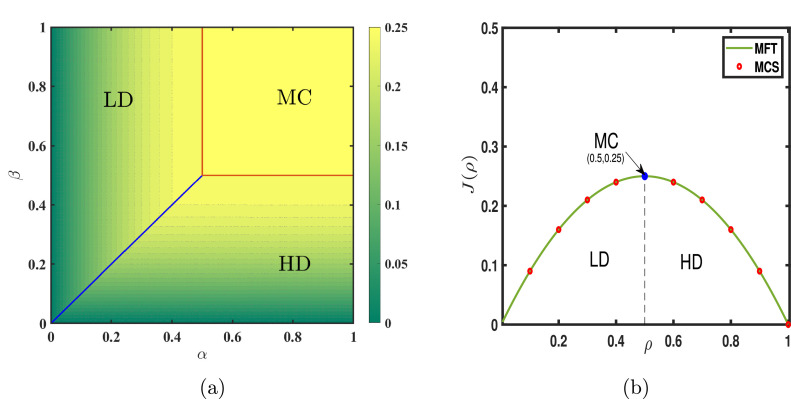
(a) Phase diagram for TASEP with open boundary conditions comprises three distinct phases: LD, HD, and MC. The contour lines supported by a color bar represent the variation of current with respect to ρ. Solid blue and red lines depict the boundaries through which first and second order transitions take place. (b) A fundamental diagram which gives the current-density relation (J=ρ(1−ρ)) displaying agreement between mean-field theory and Monte Carlo simulations obtained for random sequential update rule with an open system.

Fig. 7 illustrates the density profiles for each phase and the phase diagram in the α−β plane is presented in Fig. 8(a). On the transition line between the low density and high density phases, the entry and exit rates are equal, leading to a shock that separates the low-high density segments. This shock is delocalized and randomly moves throughout the lattice. The variation of density across this transition line is discontinuous resulting in a first order transition. However, the transition from low density as well as high density phase to maximal current phase through the lines α=1/2 and β=1/2, respectively, is characterized by a continuous change in density as well as current, therefore a second order phase transition occurs.

## Analysis of TASEP with periodic conditions

We now discuss the effect of the periodic boundaries on the stationary properties of the system. Contrary to open systems, the number of particles N remains constant. Moreover, owing to the hardcore exclusion principle, N is bounded above by L. Since the dynamics in the bulk is similar to the open case, the master equation given by Eq. (3) remains unaltered. However, for the boundaries, the equations are expressed as

(38) $$\begin{array}{cc} \frac{d\langle \tau_{1}\rangle }{dt} & =\langle \tau_{L}\left(1-\tau_{1}\right)\rangle -\langle \tau_{1}\left(1-\tau_{2}\right)\rangle ,\\ \frac{d\langle \tau_{L}\rangle }{dt} & =\langle \tau_{L-1}\left(1-\tau_{L}\right)\rangle -\langle \tau_{L}\left(1-\tau_{1}\right)\rangle . \end{array}$$

Here, we leave the discussion on transient solutions to the reader which can be contemplated analogously as done for open boundary conditions. The only change that arises in Eqs. (14)–(24) is the initial condition. Owing to the fact that the number of particles is conserved in case of periodic boundaries, the initial function g(x) must be chosen such that $\int_{0}^{1}g\left(x\right)\;dx=N/L$. For convenience, the value of $\frac{N}{L}$ is denoted by n.

Using similar mean-field treatment as performed in the case of open boundaries, the master equations for the boundaries (Eq. (38)), in steady state reduces to

(39) ρ(0)=ρ(1).

Additionally, due to the number of particles being conserved,

(40) $$\int_{0}^{1}\rho dx=n,$$

must hold.

**Fig. 9:**
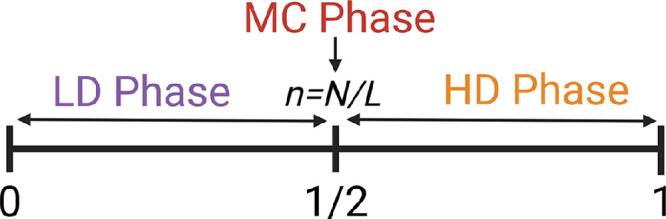
Phase line for the system with periodic boundaries representing the three phases. The particle density is regulated by the parameter n=N/L and is given by ρ=n. For the LD phase, ρ<1/2, in HD phase, ρ>1/2, and in MC phase, ρ is exactly equal to 1/2[37].

The particle density is controlled by the parameter n and can portray one of the three distinct phases: LD, HD, and MC depending on the number of particles. This dependence of the phases on n, where 0≤n≤1, can be represented in the form of a phase line (see Fig. 9). For n<1/2, the LD phase prevails, whereas when n>1/2, the system is in the HD phase. If n=1/2, i.e., the system is exactly half-filled, the MC regime occurs.

Till now, we have analyzed the TASEP with both open and periodic boundaries through mean-field approach and obtained their steady state properties. To validate these theoretical outcomes, we first use Monte Carlo simulations with a random sequential update rule, followed by the parallel update.

## Random sequential update

**Fig. 10:**
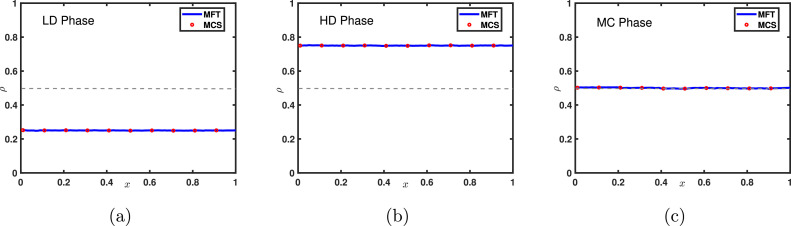
Density profiles for the system with periodic boundaries where (a) n=0.25, (b) n=0.5, and (c) n=0.75. Solid lines are the theoretical outcomes whereas symbols denote MCS results. The lattice length is taken to be L=1000. These profiles remain the same for both the random sequential and parallel updating rules.

**Fig. A.1:**
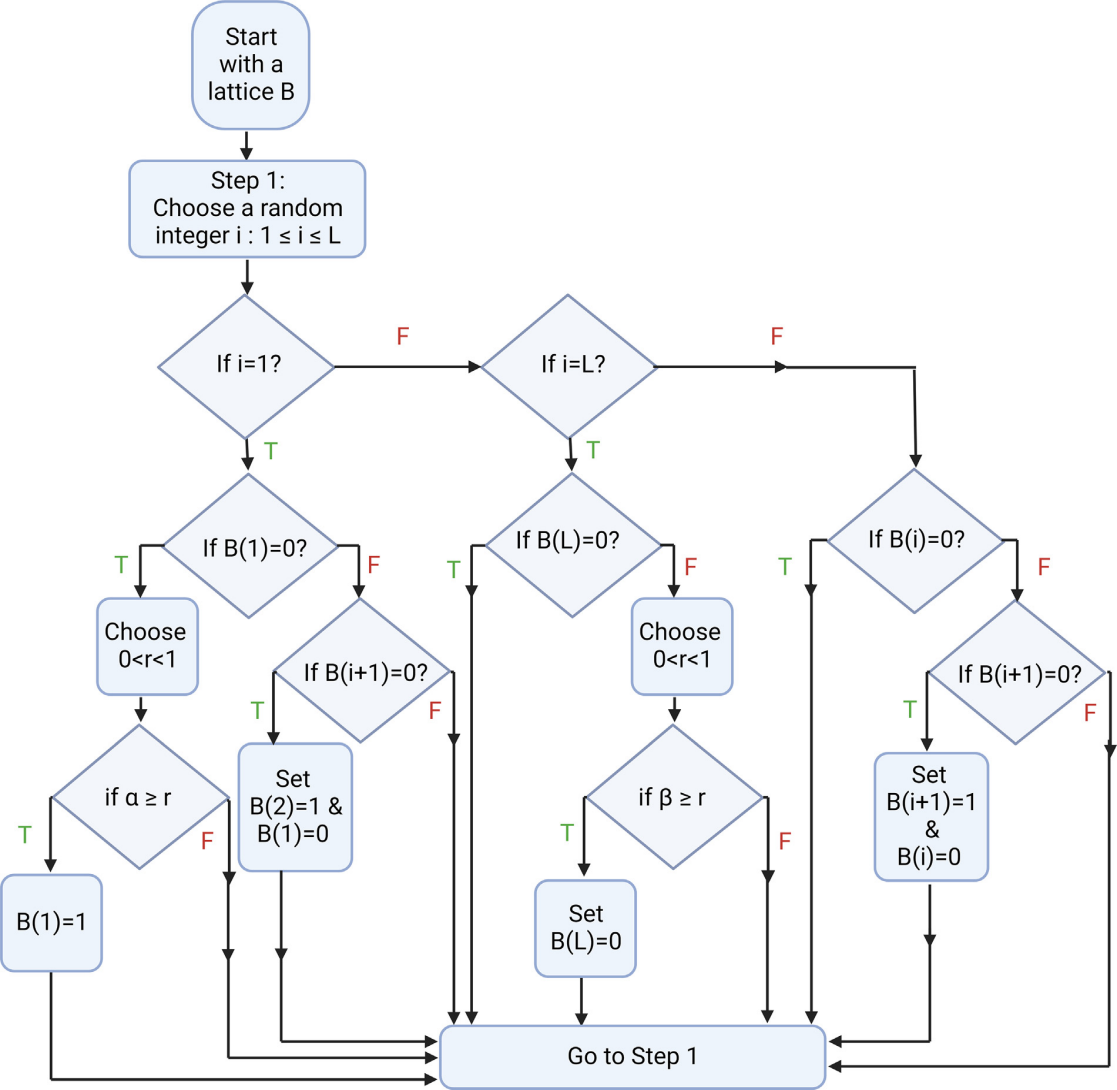
Flow chart to perform Monte Carlo simulation for TASEP with random sequential update rule. The variable r is a uniformly distributed random number between 0 and 1. After repeating the above process for a long time, the initial 5% of the iteration are neglected to ensure the onset of steady state and the average particle density is calculated by taking time average over an interval of 10L[37].

To explore the stationary system properties such as particle density, phase transitions, and density profiles, we implement Monte Carlo algorithm and compare the results generated with those of the above analyses. The flow chart of the Monte Carlo simulation (MCS) for the random update procedure is depicted in Appendix (Fig. A.1). The simulations are carried out for $L\times 10^{5}$ time steps and the first 5% of the observations are ignored to establish the existence of the stationary state. It is evident from Fig. 7, Fig. 8 and 10 that the results obtained through MCS match very well with the outcomes of the mean-field framework for all the three phases (LD, MC and HD) in case of both open and periodic boundaries [1]. This agreement between the theoretical findings and MCS is not only for the phase boundaries but also in terms of all other stationary properties such as density profiles and current. It indicates that the random update procedure does not incorporate any sort of correlations into the system which are also ignored by the mean-field theory.

## Parallel update

**Fig. 11:**
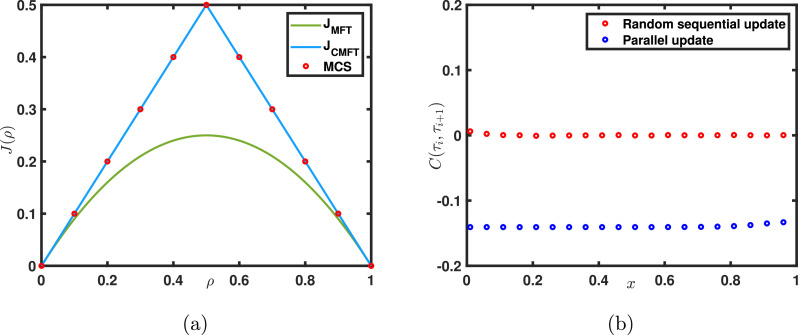
(a) Steady state current-density relation obtained through mean-field theory, cluster mean-field theory, and Monte Carlo simulation using parallel update rule. Results procured through MCS and cluster mean-field theory are in excellent agreement. (b) Correlation function plots for both random and parallel update rules.

**Fig. A.2:**
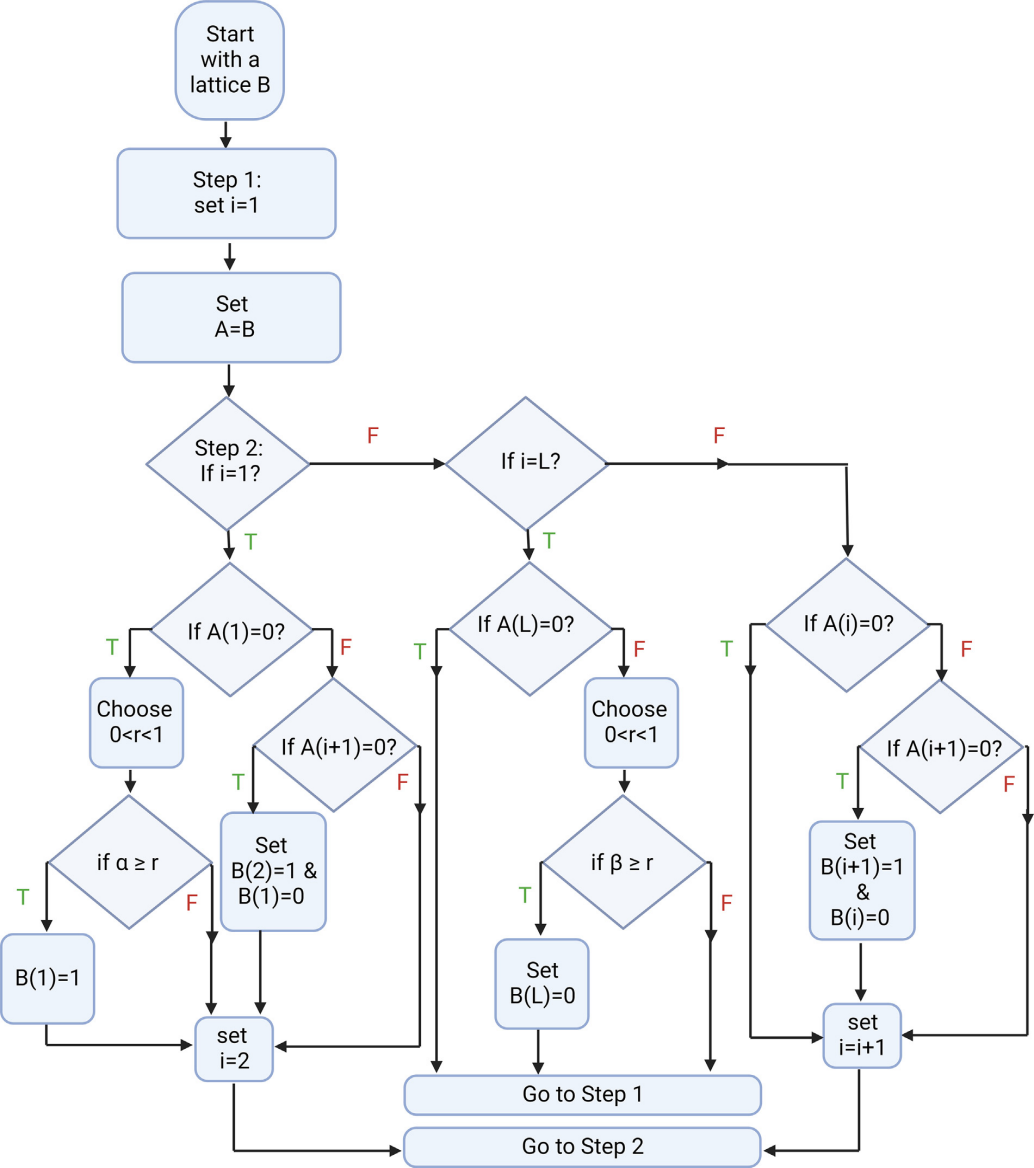
Flow chart to perform Monte Carlo simulation for TASEP with parallel update rule. The variable r is a uniformly distributed random number between 0 and 1. After repeating the above process for a long time, the initial 5% of the iteration are neglected to ensure the onset of steady state and the average particle density is calculated by taking time average over an interval of 10L[37].

In this section, we explore the system properties for the parallel update rule where at each time step, all the sites are updated simultaneously. It can be easily observed that the change in updating rule has no influence at macroscopic level i.e., the master equations given by Eqs. (2)–(4) are unaltered. This implies that the results obtained through mean-field approximation are independent of the update rule considered. However, this update procedure differs from the random sequential update rule on a microscopic level, and hence it may impact the system properties. If Monte Carlo simulations (see Appendix: Fig. A.2) are utilized to study the stationary properties of the system specifically the particle current, the outcomes of MCS, and mean-field theory do not match as shown in Fig. 11(a). Moreover, the current-density relationship as acquired from MCS deviates both quantitatively and qualitatively from that of random sequential procedure. The characteristics of the fundamental diagram such as the shape and the critical point for parallel update rule significantly differ in comparison to the random sequential update rule. Therefore, it is irrational to expect the mean-field theory, which has an excellent agreement with random sequential update, to work with parallel update as well. Since the correlations are completely neglected in mean-field theory, our first attempt is to identify the presence of correlations in the system. As confirmed from Fig. 11(b) the parallel updating rule incorporates significant correlations into the system.

**Fig. 12:**
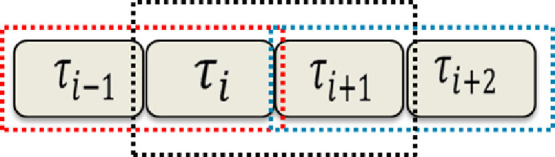
Representation of a two-cluster approximation for a cluster of four sites.

To find theoretical results for the parallel update rule, we need to employ a theory that incorporates the effect of correlations. In this direction, an improvised mean -field theory, known as two-site cluster approximation has been exploited [29], which provides the exact results for the system. Note that the naive mean-field theory discussed in section 5.1 can also be referred to as one-site cluster mean-field theory. The two-site cluster approximation assumes that a larger cluster is factorised in clusters of two (see Fig. 12) as follows,

(41) $$P\left(\tau_{1},\cdots ,\tau_{L}\right)\propto P\left(\tau_{1},\tau_{2}\right)P\left(\tau_{2},\tau_{3}\right)\cdots P\left(\tau_{L-1},\tau_{L}\right).$$

The four possible configurations that a two-site cluster can take are {0,0}, {0,1}, {1,0}, {1,1}. Let the probability associated with them be denoted by P(00), P(01), P(10), and P(11) such that their mutually exclusive and exhaustive nature yields

(42) P(00)+P(01)+P(10)+P(11)=1.

Under the Kolmogorov consistency conditions [38],

(43) $$\begin{array}{cc} P(1) & =P(10)+P(11)=\rho ,\\ P(0) & =P(00)+P(01)=1-\rho , \end{array}$$

that yields the following symmetry relations:

(44) $$\begin{array}{cc} & P(00)=1-\rho -P(10),\\ & P(11)=\rho -P(10),\\ & P(10)=P(01). \end{array}$$

One can write the master equation for P(1,0) in terms of four-point correlators as

(45) $$\begin{array}{ccc} \frac{dP(1,0)}{dt} & = & P(1000)+P(1001)+P(0110)+P(1110)\\ & & -(P(0100)+P(1100)+P(0101)+P(1101)). \end{array}$$

Utilizing Eqs. (42)–(44), the above master equation reduces to a quadratic equation

(46) $${\left(P\left(10\right)\right)}^{2}-P\left(10\right)+\rho \left(1-\rho \right)=0.$$

Solving this quadratic equation yields

(47) $$P\left(10\right)=\frac{1}{2}\left[1-\sqrt{1-4\rho (1-\rho )}\right],$$

and the corresponding particle current is given by J=P(10) which can be simplified as

(48) $$J\left(\rho \right)=\left\{ \begin{array}{cc} \rho ; & \rho \leq 1/2\\ 1-\rho ; & \rho \geq 1/2. \end{array}\right.$$

It can be easily verified that the particle current is higher when the correlations are not ignored i.e., J=P(10)≥P(1)P(0). Moreover, a comparison between random sequential and parallel update reveals that although the expressions of density for both the rules remain the same, the expressions for the current differs. It can be seen from Fig. 11 that particle flow is enhanced for parallel update rule. This implies that a particle-hole attraction is present for the parallel update case. Thus the two-point correlations can not be ignored in this case, highlighting the choice of update rule in the definition of TASEP.

Now, we explore the stationary properties of the system when incorporated with the boundary conditions: open and periodic under parallel updating rule.

### Open boundary conditions

As discussed earlier in the section. 5.3, when the density solution is less than 1/2, the corresponding phase is termed as LD phase while for ρ>1/2, we have HD phase and when ρ is exactly equal to 1/2, the system displays MC phase. To construct the stationary phase diagram, the extremal principle [39] is utilized which states that

(49) $$J=\left\{ \begin{array}{cc} \max J(\rho ); & \rho (1)<\rho <\rho (0)\\ \min J(\rho ); & \rho (0)<\rho <\rho (1). \end{array}\right.$$

#### Low density phase

In this phase, the criteria that ρ<1/2 and the expression for current given by Eq. (48) together imply that the bulk current is given by J(ρ)=ρ. The continuity of current in the system guarantees that the bulk current is equal to the current entering the first site, which gives

(50) $$\alpha \left(1-\rho \right)=\rho \Rightarrow \rho =\frac{\alpha }{1+\alpha }.$$

Since the density in this phase remains less than 1/2, we have α<1.

#### High density phase

The existence of this phase requires ρ>1/2 and the corresponding bulk current from Eq. (48) yields J=1−ρ. Using a similar approach as done for the LD phase, the expression for density in the HD phase can be calculated as

(51) $$\rho =\frac{1}{1+\beta }.$$

Moreover, the condition ρ>1/2 implies that β remains less than 1.

#### Maximal current phase

In this situation, the particle density becomes independent of the two boundary conditions, and the bulk density is solely given by 1/2. At this value, the current attains its maximum point which is 1/2. As a consequence of the current continuity throughout the lattice, this phase exists when

(52) α=β=1.

The above examination reveals that for the parallel updating rule, the particle density at the extreme sites is given as

(53) $$\rho \left(0\right)=\frac{\alpha }{1+\alpha }\;\text{and}\;\rho \left(1\right)=\frac{1}{1+\beta }.$$

Since ρ(0) is always less than ρ(1), the extremal principle given by Eq. (49) simplifies to

(54) $$J=\min\left\{\frac{\alpha }{1+\alpha },\;\frac{1}{2},\;1-\frac{1}{1+\beta }\right\}.$$

Utilising the fact that the in LD and HD phase, the bulk current is governed by the densities of the first and the last site, respectively, we have the following existential conditions for each phase:

(55) $$\rho =\left\{ \begin{array}{cc} \frac{\alpha }{1+\alpha }; & \alpha <\min\{\beta ,1\}\\ \frac{1}{1+\beta }; & \beta <\min\{\alpha ,1\}\\ \frac{1}{2}; & \alpha =\beta =1. \end{array}\right.$$

**Fig. 13:**
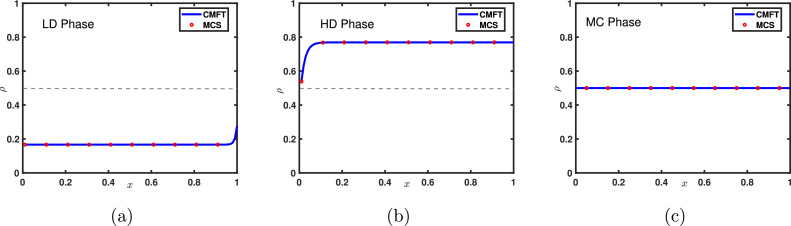
Density profiles for the system with open boundaries and parallel update rule where (α,β)= (a) (0.2,0.6), (b) (0.5,0.3) and (c) (1,1). The length of the lattice is equal to 1000. Solid lines correspond to cluster mean-field results and symbols denote Monte Carlo simulations.

**Fig. 14:**
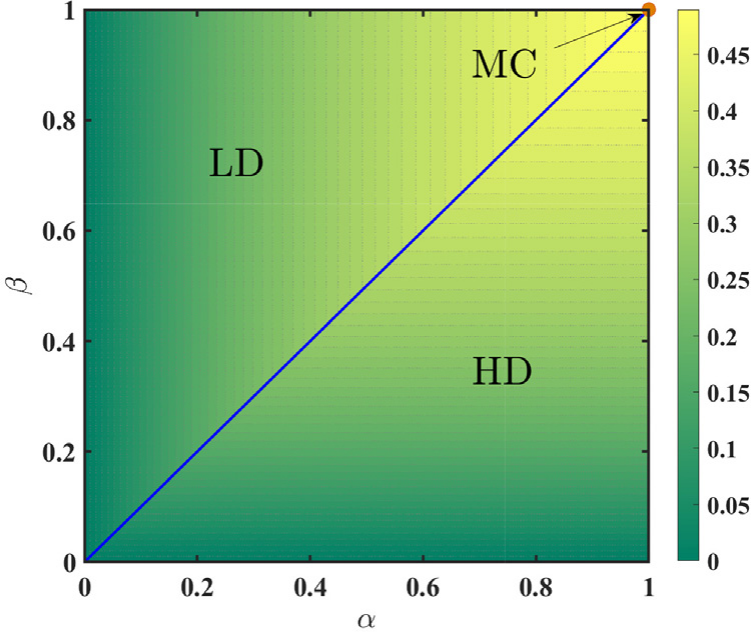
Phase diagram for TASEP with open boundaries under parallel update rule in α−β parameter space consists of three distinct phases: LD, HD, and MC. Solid blue lines describe the line of first-order transition. The contour lines represent the currents at the respective point whose intensity is given by the accompanying color bar.

It can be easily deduced from the above equation that a first-order transition takes place across the line α=β from LD to the HD phase. It is noteworthy that, here MC phase occurs only at a single point, in contrast to the random sequential update rule. The corresponding density profiles and the phase diagram is presented in Figs. 13 and 14, respectively.

In the case of periodic boundaries, the characteristics of the phase line as well as the density profiles (see Figs. 9 and 10) are the same as that obtained through MFT and random sequential update rule, which reveals three stationary phases: LD, MC, and HD, characterized by the density ρ=n and current is given by Eq. (48).

## Numerical Technique

We provide an alternative method to obtain solutions for the second order partial differential equation given by Eq. (11) for mean-field theory which may be difficult to solve analytically. Time derivative is kept intact in the system and the density solutions at a steady state are procured in the limit t (number of timesteps) →∞. The forward in time and central in space (FTCS) scheme is utilized to obtain the numerical solutions as follows:

(56) $$\rho_{i}^{j+1}=\rho_{i}^{j}+\frac{\epsilon ▵t}{2}\left(\frac{\rho_{i+1}^{j}-2\rho_{i}^{j}+\rho_{i-1}^{j}}{▵x^{2}}\right)+▵t\left(\frac{\rho_{i+1}^{j}-\rho_{i-1}^{j}}{2▵x}\right)\left(2\rho_{i}^{j}-1\right).$$

The notation $\rho_{i}^{j}$ denotes the numerical approximation at the point $\left(x_{i},t_{j}\right)$. At the boundaries, Eqs. (8) and (10) are explicitly utilized to attain

(57) $$\begin{array}{cc} \rho_{1}^{j+1} & =\rho_{1}^{j}+▵t\left(\alpha \left(1-\rho_{1}^{j}\right)-\rho_{1}^{j}\left(1-\rho_{2}^{j}\right)\right),\\ \rho_{L}^{j+1} & =\rho_{L}^{j}+▵t\left(\rho_{L-1}^{j}\left(1-\rho_{L}^{j}\right)-\beta \rho_{L}^{j}\right). \end{array}$$

Here, the space variable is discretized as ▵x=1/L and the choice of ▵t obeys $▵t/▵x^{2}\leq 1$ which is the stability criteria of the above numerical scheme.

## Summary and outlook

To summarize, we discussed the complicated non-equilibrium transport phenomena on one channel, in particular driven diffusive systems that have influenced stochastic models with rich collective features. These systems are modelled as totally asymmetric simple exclusion process which is provided with suitable boundary condition. The different techniques to comprehend the system properties have been elaborated with a focus on understanding the stationary state of the system.

The collective evolution of the system is viewed in a form of a set of master equations which is reduced to a continuum equation using an appropriate approximation. Here, we concentrate on the mean-field methods and ignore few or all correlations among the particles to reduce the many-body system into a one-body system. We have detailed two different methods to analyze the resulting continuum equation obtained using the mean-field approach. The first method relies on theoretical techniques and yields the analysis of system properties in the stationary state. The theoretical calculations of the totally asymmetric simple exclusion process with two types of boundary conditions have been provided. The second method relies on the numerical scheme, mainly the finite-difference method.

The two different types of boundary conditions are discussed: open boundaries and periodic boundaries. Both the categories have been analyzed intensively using the theoretical approach and both transient, as well as steady state solutions, have been provided. We have seen the rich system properties that appear in a totally asymmetric exclusion process equipped with open boundaries. The simple dynamics even result in the phase transitions that are regulated by the boundary conditions. In the case of periodic boundaries, the system properties are controlled by the number of particles.

Furthermore, we detailed random sequential as well as parallel update schemes and discussed the algorithm to capture the system dynamics via simulations. In particular, we elaborated Monte Carlo simulations with regard to the update procedure in use. We also briefly touched upon the correlations that arise in the system depending on the update procedure. In particular, parallel updates introduce correlations which, if ignored, affect the outcomes of theoretical and numerical methods. Therefore, an appropriate mean-field approximation, namely the cluster mean-field approach, is utilized that not only theoretically solves the system and provides insight into its qualitative properties, but also agrees with the quantitative outcomes of the system.

Moreover, we have also discussed how update procedures affect the properties which can be overlooked by the approximation if it is incompatible. It was seen that for both random sequential and parallel updates, the resulting continuum equation is identical when the simple mean-field theory is used. However, the technique fails in parallel update whereas the results are exact for the random sequential update. This supports the fact that although the dynamics are the same at the micro level, they significantly affect the collective behaviour and results differ.

## Declaration of Competing Interest

The authors declare that no competing financial interests or personal relationships exist.

## Data Availability

No data was used for the research described in the article.

## Appendix

Monte Carlo simulation is one of the popular and efficient methods to validate the theoretical finding obtained through various multi-particle systems [35], [36]. The following flowcharts summarize the Monte Carlo simulation for both random sequential and parallel update procedures.
